# Acute SARS-CoV-2 viral load and systemic inflammation are associated with neuropsychiatric and musculoskeletal symptoms in long COVID

**DOI:** 10.1371/journal.pone.0346978

**Published:** 2026-04-15

**Authors:** Uzair Abbas, Rabeel Nawaz Laghari, Ishfaque Ahmed, Usama Abdul Musawwir, Hina Riaz, Khadija Anwar, Niaz Hussain, Muhammad Mubeen, Mahtab Khan, Muhib Ullah Khalid, Shizrah Ashraf

**Affiliations:** 1 Department of Physiology, Dow University of Health Sciences, Karachi, Pakistan; 2 Department of Medicine, Indus Medical College Hospital, Tando Mohammad Khan, Pakistan; 3 Sindh Infectious Diseases Hospital and Research Center, Dow University of Health Sciences, Karachi, Pakistan; 4 Dow International Medical College, Dow University of Health Sciences, Karachi, Pakistan; 5 Bilawal Medical College, Liaquat University of Medical & Health Sciences, Jamshoro, Pakistan; 6 Fauji Foundation Hospital, Rawalpindi, Pakistan; Johns Hopkins School of Medicine: The Johns Hopkins University School of Medicine, UNITED STATES OF AMERICA

## Abstract

**Background:**

Long after recovery from acute-COVID illness, many patients show persistent multi-organ dysfunction consistent with Long COVID. Biochemical profile and measurements of inflammatory markers in these individuals can help to understand the underlying pathophysiology. This study aims to evaluate biochemical markers and their association with symptoms of Long COVID. We, in a retrospective analysis, also examined whether the Long COVID symptom persistence is associated with the SARS-CoV-2 viral load documented during the acute infection.

**Methods:**

A total of 300 participants with previously diagnosed mild COVID-19 were recruited at 10 months post-infection. Brief clinical history was taken based on persistent symptoms after COVID-19 and categorized as Long COVID (n = 177) and controls group (n = 123) based on WHO defined criteria. Biochemical parameters in blood like complete blood count (RBC and WBC indices) were compared between the groups. Other measurements including inflammatory markers such as IL-6, IL-10, ferritin and C-reactive protein along with electrolytes, vitamin D3 and B12, and lipid profile, were also compared. SARS-CoV-2 viral load was assessed retrospectively. Data was analyzed through SPSS v.26.

**Results:**

The findings of our study revealed that 59% (177) of individuals had symptoms of Long COVID. The most frequently reported symptoms of Long COVID were related to neuropsychiatry (35%), followed by musculoskeletal system (32.2%). The Hemoglobin, RBC counts and MCHC were decreased in Long COVID as compared to control group (p < 0.05). While Lymphocytes, IL-6 and ferritin levels were raised in Long COVID group (p < 0.05). In multivariable logistic regression analyses adjusted for age and sex, neuropsychiatric symptoms were independently associated with higher lymphocyte counts (aOR 1.19, 95% CI 1.12–1.51), IL-6 (aOR 1.16, 95% CI 1.10–1.86), ferritin (aOR 1.42, 95% CI 1.10–1.53), and vitamin D deficiency (aOR 1.45, 95% CI 1.22–2.01). Musculoskeletal symptoms were strongly associated with vitamin D deficiency (aOR 2.30, 95% CI 1.20–4.50) and ferritin levels (aOR 0.98, 95% CI 0.97–0.99). Moreover, higher SARS-CoV-2 viral load (CT ≤ 20) during acute infection was also associated with neuropsychiatric and musculoskeletal symptoms of Long COVID.

**Conclusion:**

We identified Long COVID in 59% of the participants, the highest reported percentage in studies in the middle-aged individuals. Compared to controls, we found differential biochemical markers in the Long COVID group indicating a different metabolic status in these individuals. Moreover, the association of raised inflammatory markers at ten months follow up and acute-phase SARS-CoV-2 viral load were also seen to be associated with musculoskeletal and neuropsychiatric symptoms of the Long COVID. These significant clinical and biochemical changes warrant thorough monitoring and follow-ups for extended time.

## Background

The coronavirus disease (COVID-19) pandemic was a public health concern because of its expansion worldwide, with 677 million cases and 6.9 million deaths reported worldwide to July 2024 [1]. Pakistan with its 220 million population, reported approximately 1.5 million cases and 31,000 COVID-19 related deaths [2]. While most individuals recover from the acute infection, a sizable portion of individuals report long-term varying symptoms lasting from weeks to several months following apparent recovery from the acute infection [3]. This condition is known as “Long COVID” as defined by World Health Organization (WHO) occurring typically after 3 months of acute COVID-19 infection [4].

Risk for Long COVID appears higher in females, advanced age, individuals with co-morbid conditions as well as those who experienced more symptoms during the acute infection (≥5 symptoms) [5]. Long COVID may involve multiple organ systems including neurological, respiratory, musculoskeletal, and reproductive systems with common manifestations such as fatigue, cognitive impairment, dyspnea, sleep disturbances, and musculoskeletal pain [6–9]. The underlying pathophysiology contributing to these symptoms is complex and includes micronutrient deficiency, autonomous dysfunction, chronic inflammation, immune dysregulation and occult viral persistence [10].

Emerging evidence suggests that inflammatory markers such as interleukin-6 (IL-6), C-reactive protein (CRP), and ferritin may remain elevated months after acute infection [11]. Micronutrient deficiencies have also been implicated in persistent symptomatology [12]. However, much of the available literature focuses on survivors of severe or critical illness, and data among individuals who experienced mild disease particularly in middle-aged populations remain limited.

The relationship between acute-phase viral load, reflected by reverse transcription polymerase chain reaction (RT-PCR) cycle threshold (Ct) values, and the subsequent development of Long COVID remains an area of ongoing investigation. Although higher viral loads have been associated with severe acute COVID-19 [13], evidence regarding their association with persistent symptoms following mild infection is scarce. Clarifying whether acute viral burden influences long-term symptom profiles may enhance understanding of Long COVID pathogenesis.

Understanding the biological determinants of persistent symptom clusters following mild COVID-19 is clinically important, as most infected individuals do not require hospitalization yet may experience prolonged morbidity. Identifying inflammatory and micronutrient alterations associated with specific symptom phenotypes may contribute to improved risk stratification and inform future mechanistic and interventional studies.

Therefore, this study aimed to estimate the frequency of Long COVID among middle-aged individuals recovering from mild infection and to determine whether inflammatory biomarkers, vitamin D and vitamin B12 levels, and acute-phase viral load were independently associated with dominant symptom clusters at ten months post-infection.

## Methodology

### Study description

In this study, we enrolled 300 adults from Dow University Hospital (DUH), Karachi Pakistan from February 2023 to August 2024. The participants were matched for age and gender. Sample size was estimated from a reference study [14].

### Ethical statement

The study adhered to the ethical principles of the Declaration of Helsinki and received approval from Dow University of Health Sciences (DUHS) Institutional Review Board (IRB). The reference number is IRB/DUHS/Approval/2022–236. A written informed consent was obtained from all participants before enrollment.

### Recruitment of participants

#### Inclusion criteria.

We enrolled 300 participants with age ranging from 30 to 60 (middle-aged), and either male or female gender. Individuals with previous history of a positive COVID-19 PCR attending follow-up clinics in Outpatient Departments (OPDs) of Medicine or Infectious Disease of Dow University Hospital were included. Following the World Health Organization criteria for disease categorization, only the individuals who had asymptomatic or mild COVID-19 illness were included in the study [15]. We also used another approach to recruit participants. The lists of PCR testing for SARS-CoV-2 were collected from Dow Diagnostic Research and Reference Laboratory (DDRRL) retrospectively. Candidates meeting eligibility were contacted, briefed about the study, and invited to attend the outpatient clinic for screening and enrollment.

#### Exclusion criteria.

We excluded participants with a history of severe-critical COVID-19, hospitalized for COVID-19, participants with a positive SARS-CoV-2 PCR less than 9 or more than 11 months from date of recruitment. We also excluded individuals who had diabetes or other metabolic disorders, recent or chronic viral infections (hepatitis B or C, HIV, or tuberculosis), and pregnancy. Individuals receiving hormone therapy, diuretic antihypertensives, immunosuppressive agents, or NSAIDs were also excluded. Those who did not consent to participate (n = 37; either in clinics or phone calls) or those who got COVID-19 infection more than once were also excluded.

### Data collection

At enrollment, with help of a structured questionnaire, data from all participants including demographics (age, gender), acute-phase symptoms of COVID-19, and current presenting complaints related to long COVID were collected. Participants were further categorized into Long COVID (n = 177) and No Long COVID-19 or the controls (n = 123) based on their clinical examination.

### Definition of long COVID

Long COVID was defined according to World Health Organization criteria as the presence of new or persistent symptoms occurring at least 3 months after confirmed SARS-CoV-2 infection, lasting for at least 2 months, and not explained by alternative diagnoses [4].

### Symptom assessment

Symptoms were assessed using a structured questionnaire administered by trained physicians. Symptoms were collected by organ system: central nervous system (headache, cognitive complaints/“brain fog,” insomnia, tremors); cardiovascular (palpitations); respiratory (dyspnea, cough, chest pain); musculoskeletal (asthenia, myalgia, weakness, fatigue, arthralgia); gastrointestinal (abdominal pain); ear-nose-throat (anosmia, dysgeusia); and reproductive (dysmenorrhea, irregular cycles, erectile dysfunction).

Participants were classified into dominant symptom clusters based on their most prominent ongoing symptom at the time of evaluation.

### Assessment of hematological markers

Blood sample (10 mL) of each participant was collected and processed at institutional laboratory. The panel included complete blood count with erythrocyte and leukocyte indices; fasting lipid profile (total cholesterol, HDL-C, LDL-C, triglycerides); urea and creatinine; electrolytes (Na ⁺ , K ⁺ , Cl ⁻ , HCO₃⁻); inflammatory markers (C-reactive protein, interleukin-6, interleukin-10, ferritin); vitamin B12; and 25-hydroxyvitamin D3. The reference for normal ranges of laboratory parameters was provided by the laboratory [16–18].

Laboratory investigations were performed at Dow Diagnostic, Research and Reference Laboratory services using standardized automated analyzers. Hematological parameters were analyzed using automated hematology analyzers based on impedance and flow cytometry principles. IL-6 and IL-10 were measured using enzyme-linked immunosorbent assays, while CRP and ferritin were assessed using automated immunoassays. Biochemical parameters were measured using enzymatic colorimetric methods, and electrolytes were analyzed using ion-selective electrode techniques. Vitamin D and vitamin B12 levels were determined using chemiluminescent immunoassays. All analyses were conducted according to manufacturer protocols with internal quality control measures.

### Assessment of acute-phase viral load

For viral load analyses, only participants who were tested at clinical laboratory of our institute were included. Retrospective data in terms of cycle-threshold (Ct) value for viral load was retrieved from the laboratory record. Ct values were used as an inverse surrogate marker of viral load, where lower Ct values indicate higher viral RNA concentration. Following were the three groups for categorization of viral load base on the Ct value: ≤ 20, 20.1 to 30 and >30 as described earlier [8]. Consideration of records was limited only to the RT-PCR assays performed within 72 hours of COVID-19 symptoms onset.

The study was carried out between a time when the omicron variants were in circulation in Pakistan [19], but the variant of concern during active infection of the recruited participants could not be determined through previous laboratory reports as genomic sequencing was not routinely performed during that period.

### Data analysis

Analyses were performed using Statistical Packages for Social Sciences (SPSS v26.0) and GraphPad PRISM (v.8.1). Mean and frequencies were calculated through simple statistical analysis. Continuous variables were assessed for normality using the Shapiro–Wilk test. Non-normally distributed variables were analyzed using non-parametric tests. Categorical variables were compared using the chi-square test.

To evaluate independent associations between laboratory parameters and dominant Long COVID symptom clusters, multivariable logistic regression analyses were conducted. Separate models were constructed for dominant symptoms of Long COVID. Adjusted odds ratios (aOR) with 95% confidence intervals (CI) were calculated after controlling for age and sex. Spearman correlation analysis was performed to evaluate associations between acute-phase Ct values and inflammatory biomarkers. A two-sided p-value <0.05 was considered statistically significant.

## Results

### A. Demographic and clinical characteristics

**Fig 1:**
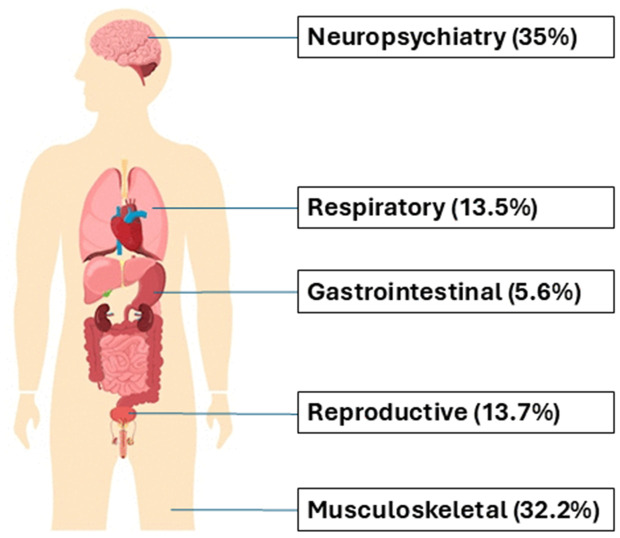
Depiction of Long COVID symptoms in study participants (n = 177). The figure shows the percentage of Long COVID symptoms based on physiological systems. Highest percentage of symptoms were neuropsychiatry followed by musculoskeletal.

The mean age of participants was 44.69 ± 9.11 years with 143 (47.66%) of females. The demographic and clinical characteristics of study groups have been shown in Table 1. Overall, we found 177 (59%) of study participants experiencing long COVID and related complaints were assessed with respect to the organ system (Fig 1). The most common symptoms were related to neuropsychiatry (62/177; 35% which included confusion, insomnia, problem in decision making, recurrent headaches, and problem in memory retention), followed by musculoskeletal (57/177; 32.2% which included myalgia, muscle weakness, fatigue and arthralgia), and respiratory (24/177;13.5% which included dyspnea, cough, recurrent flu and chest pain). The remaining participants were facing other symptoms including reproductive organ systems (including dysmenorrhea, irregular menstrual cycle in females and erectile dysfunction in males) and gastrointestinal tract.

**Table 1:** Demographic characteristics of study groups (n = 300).

Variables		Long COVID (n = 177)	Controls (n = 123)	p value
**Age (years)**	Mean (SD)	45.74 ± 11.48	43.25 ± 14.92	0.477
**Gender**	Female	82 (46.4%)	61 (49.59%)	0.58
	Male	95 (53.6%)	62 (50.41%)	
**BMI**	Kg/m^2^	22.1 ± 2.3	23.5 ± 2.7	0.783
**COVID-19 vaccine**	Yes	176 (99.4%)	123 (100%)	0.761

Statistical analysis between groups was performed using either Mann-Witney test, or the chi square test. *Differences are significant at p < 0.05 at 95% CI.

### B. Comparison of hematological and biochemical markers between long COVID-19 and control groups

#### a. Red blood cell counts and indices:

**Fig 2:**
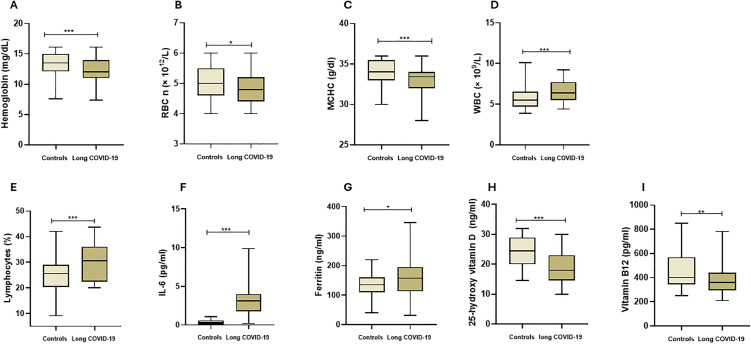
Comparison of biochemical and inflammatory markers in study participants (n = 300). The figure shows the comparison of A) Hemoglobin (Hb), B) RBC count, C) MCHC; D) WBC count, E) Lymphocyte percentage, F) IL-6, and G) Ferritin, H) Vitamin D, and I) Vitamin B12 between Long COVID (n = 177) and Controls (n = 153). The box and whisker plots show comparison of median values of variables compared through Mann-Witney test. RBC = Red Blood Cells; MCHC = Mean corpuscular hemoglobin concentration; WBC = White Blood Cells; IL-6 = Interleukin-6.

The complete blood count included hemoglobin (Hb), red blood cell (RBC) count, mean corpuscular volume (MCV), hematocrit (HCT), and mean corpuscular hemoglobin concentration (MCHC). We found a lower level of Hb (p < 0.001), RBC count (p = 0.034) and MCHC (p < 0.001) in the Long COVID group as compared to controls (Fig 2A-C). HCT and MCV were indistinguishable among study groups (S1 Table). Sex based differential RBC count, Hemoglobin levels and hematocrit values are presented in S2 Table.

#### b. White blood cells counts and inflammatory markers:

Total and differential leukocyte counts were analyzed among study groups. Long COVID demonstrated significantly higher total WBC count when compared to the control group (p < 0.001). We also found a higher lymphocyte percentage in Long COVID group (p = 0.001; Fig 2D-E). The difference in Neutrophils, Eosinophils, Basophils or Monocytes remained insignificant among the study groups (p > 0.05; S1 Table). While comparing inflammatory markers, we found elevated levels of IL-6 (p < 0.001) and ferritin levels (p = 0.013; Fig 2F-G) in Long COVID group as compared to control group. On the other hand, IL-10 and CRP levels demonstrated insignificant difference (p > 0.05; S1 Table).

#### c. Urea, creatinine, electrolytes and lipid profile:

Urea, creatinine, electrolytes (sodium, potassium, chloride, bicarbonate), and lipid profiles (total cholesterol, HDL, LDL, triglycerides) were also compared between the Long COVID and control groups with no statistically significant differences found (S3 Table).

#### d. Vitamin D and B12 levels:

Comparison of vitamin D and vitamin B12 demonstrated lower Vitamin D3 levels (p = 0.002) and Vitamin B12 levels (p = 0.014) in Long COVID group as compared to control group (Fig 2H-I).

### C. Association of hematological markers with long COVID-19 symptoms

Those hematological parameters which were observed to be significantly different between participants with long COVID and controls, were compared for their differential levels as per dominant Long COVID symptoms. We found higher levels of lymphocytes, IL-6, and ferritin among participants who were experiencing symptoms related to neuropsychiatry and musculoskeletal systems. However, vitamin D and B12 were found to be lowest among these individuals, compared by one-way ANOVA and post hoc Tukey’s test (S4 Table).

Further, multivariable logistic regression analyses were performed to evaluate independent associations between laboratory parameters and dominant Long COVID symptom clusters (S5 Table).

For neuropsychiatric symptoms, higher lymphocyte counts were independently associated with increased odds of symptom presentation (aOR 1.19; p = 0.02). Elevated IL-6 (aOR 1.16; p = 0.030) and ferritin levels (aOR 1.42; p = 0.002) were also significantly associated. Additionally, vitamin D deficiency (<20 ng/mL) demonstrated an independent association with neuropsychiatric symptoms (aOR 1.45; p = 0.003). Vitamin B12 levels were not significantly associated with the adjusted model (p = 0.07; S5 Table).

In the musculoskeletal symptom cluster, vitamin D deficiency showed a strong independent association (aOR 2.30; p = 0.001). Ferritin levels were modestly associated (aOR 0.98; p = 0.006), and IL-6 demonstrated a borderline inverse association (aOR 0.94; p = 0.036). Lymphocyte count and vitamin B12 were not significantly associated with musculoskeletal symptoms.

For the “other” symptom cluster, no consistent independent associations were observed. Although vitamin D deficiency reached borderline statistical significance (aOR 1.02; p = 0.04), lymphocyte count, IL-6, ferritin, and vitamin B12 were not significantly associated.

### D. Association between SARS-CoV-2 viral load and long COVID symptoms

**Fig 3:**
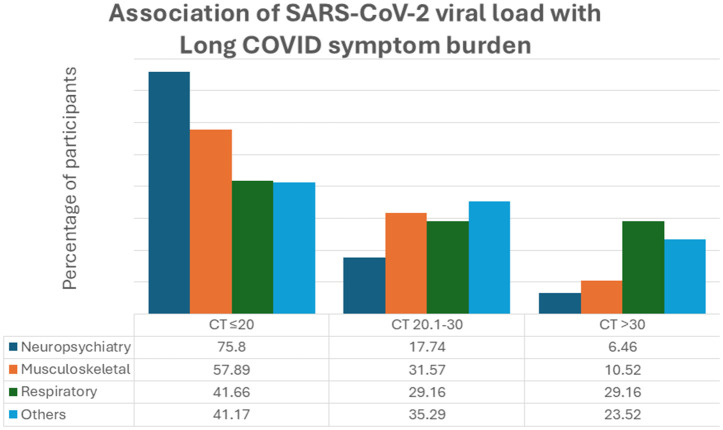
Association of SARS-CoV-2 viral load with symptoms of Long COVID (n = 177). The figure shows row percentages of participants with neuropsychiatry (n = 62), musculoskeletal (n = 57), respiratory (n = 24) and other Long COVID symptoms (n = 34).

The association between SARS-CoV-2 viral load during acute infection and Long COVID symptoms was also explored. Long COVID group was categorized into three sub-groups based on viral load according to Ct value as ≤20 (n = 104/177) 20.1 to 30 (n = 48/177) and >30 (n = 25/177) as described earlier [8]. We observed increased viral loads to be associated with neuropsychiatric and musculoskeletal systems. A larger portion of participants with neuropsychiatric symptoms (47/62; 75.8%) had viral load ≤20, while 17.74% (11/62) had viral load between CT value of 20.1 to 30 (p < 0.001). The same trend was followed by musculoskeletal symptoms in which 57.89% (33/57) had viral load of ≤20, while 31.57% (18/57) had viral load of CT value between 20.1 to 30 (p = 0.001). No differences in viral load were identified for respiratory or other symptoms related to Long COVID (Fig 3).

## E. Correlation of SARS-CoV-2 viral load and inflammatory markers

Spearman correlation analysis demonstrated a modest but statistically significant inverse association of acute-phase Ct values with IL-6 levels (rho= −0.337, p = 0.016), and ferritin levels (rho = −0.451, p = 0.001) indicating higher IL-6 and Ferritin levels among individuals with higher viral load (lower Ct values). No significant correlations were observed between Ct values and lymphocyte counts (p = 0.825).

## Discussion

In this age and gender matched case-control study of middle-aged individuals with mild or asymptomatic COVID-19 infection, we found that 59% had Long COVID at 10 months after the acute infection. Symptoms most commonly were related to neuropsychiatry, followed by musculoskeletal, respiratory, reproductive and gastrointestinal systems. It was observed that biochemical and hematological parameters, between controls and those with Long COVID, had several notable differences. Participants with Long COVID had significantly reduced hemoglobin, RBCs, with higher WBC count, lymphocytes, IL-6, and ferritin levels. Vitamin D3 and B12 were markedly lower in the Long COVID group. Our findings also showed that higher viral loads during acute infection were linked with neuropsychiatric and musculoskeletal symptoms of Long COVID.

At the 10-month follow-up, 59% of previously infected participants met criteria for Long COVID. The findings of our study align with and build on existing literature. It is broadly consistent with earlier reports indicating that roughly 40–70% of convalescent individuals exhibit persistent or newly emergent post-acute sequelae across varying follow-up intervals [20–22]. The findings of our study corroborate existing literature showing that neuropsychiatric, musculoskeletal and respiratory symptoms are the most frequently reported symptoms post-COVID, even in patients that had a mild acute infection [23]. In a comprehensive meta-analysis, which synthesized 63 studies and showed that predominant symptoms vary by follow-up interval: at 3– < 6 months, dyspnea, fatigue, sleep disturbance, and impaired concentration were common; at 6– < 9 months, effort intolerance, dyspnea, sleep disturbance, and fatigue predominated; and at 9–12 months, fatigue, dyspnea, sleep disturbance, and myalgia were most frequently reported [24].

We found hemoglobin was significantly lowered in Long COVID group along with RBC count and MCHC as compared to controls group. SARS-CoV-2 has been linked to alterations in erythrocyte morphology and rheology in severe disease, with changes persisting for up to six months after recovery [25]. Evidence in mild cases is limited. One study described lower hemoglobin, MCV, and MCHC at three months post-infection, whereas a study reported no group differences in hemoglobin, RBC count, or indices but did note morphological abnormalities relative to controls, acknowledging a small sample size [26].

Leukocyte findings in our study showed higher total WBC counts and a greater lymphocyte proportion in the Long COVID group. SARS-CoV-2 affects immune cell profiles during and after infection, and dysregulated differentials have been described. A study observed elevated neutrophils, lymphocytes, basophils, and eosinophils in recovered individuals, without differences in total WBC or monocytes versus healthy controls [26]. Conversely, lymphocytopenia during the acute phase and into follow-up has also been documented. Another study reported lymphocytopenia at six months post-acute COVID-19 illness [27]. The inflammatory markers, including IL-6, and ferritin were elevated in our study, aligning with previous studies [28,29] that reported persistent immune activation in Long COVID patients up to six months. This persistence of inflammatory markers is due to immunological dysregulation, following a viral infection. IL-6 is an important mediator of neuroinflammation and has been attributed to symptoms like brain fog, fatigue, cognitive dysfunction by disrupting the blood-brain barrier and altering neurotransmitter signaling [30]. Similarly, increased levels of ferritin indicate ongoing macrophage activation which might contribute to musculoskeletal symptoms via mitochondrial dysfunction and catabolic alterations [31]. These findings support our study and link to possible ongoing systemic inflammation even at 10 months post-acute COVID-19 infection.

While significant changes were observed in vitamin D and B12 levels in our study, micronutrient deficiency, especially that of vitamin D is significantly linked with fatigue, cognitive dysfunction and musculoskeletal symptoms [32]. An association was found between vitamin D levels and neuropsychiatric along with musculoskeletal symptoms in Long COVID. Vitamin D is vital for normal neuromuscular function and immunomodulation. Its deficiency inhibits T-regulatory cell activity, resulting in prolonged inflammation, contributing to fatigue, cognitive impairment and muscle weakness [33] which explains the associations found in our study.

While our study didn’t observe significant differences in lipid profile (total cholesterol, HDL, LDL, triglycerides), other studies have reported lipid profile alterations in Long COVID. A systematic review and meta-analysis reported significantly elevated levels of total cholesterol and triglycerides in long-COVID individuals in comparison to the controls, indicating increased atherogenicity [34]. These findings underscore the heterogeneity in lipid profiles amongst Long COVID individuals and emphasize the need for further research.

The association between SARS-CoV-2 viral load during acute infection and clinical phenotype in Long COVID remains incompletely characterized. While higher viral burden may carry prognostic value in individuals with compromised immune function, high titers are also observed in patients with mild or asymptomatic infection, limiting the potential use of viral load to serve a role as indicator of disease course [35]. We further explored if our findings have any correlation with SARS-CoV-2 viral load in acute phase. We found higher viral load in individuals with neuropsychiatric and musculoskeletal symptoms of Long COVID. Higher viral load of SARS-CoV-2 have been associated with immune activation, and a surge in cytokine levels (IL-6, TNF-α), leading to widespread inflammation and tissue injury. This elevated neuroinflammation may disrupt the integrity of blood-brain barrier, resulting in symptoms like brain fog, cognitive dysfunction and fatigue [36]. Similarly, myalgia and weakness can be attributed to inflammatory damage to the muscles and mitochondrial dysfunction [37] which might be attributed to higher viral load in initial COVID-19 illness.

In multivariable analyses, distinct biological patterns were observed across symptom clusters. Neuropsychiatric manifestations were independently associated with higher lymphocyte counts, IL-6, and ferritin levels, as well as vitamin D deficiency, suggesting a persistent inflammatory and immune activation profile. In contrast, musculoskeletal symptoms demonstrated a strong independent association with vitamin D deficiency, with a more modest contribution from ferritin and IL-6. These findings support the hypothesis that Long COVID may represent heterogeneous phenotypes characterized by differing inflammatory and micronutrient signatures rather than a uniform post-viral syndrome. Also, the observed inverse correlation of Ct values with IL-6 and ferritin supports the hypothesis that higher acute viral burden may contribute to sustained inflammatory activation [38].

## Conclusions

The significant burden (59%) of long COVID beyond 10 months of acute mild infection calls for long-term follow-up. These observations emphasize the urgent need for comprehensive post-COVID systemic monitoring, the establishment of uniform clinical management protocols and standardized treatment guidelines. Persistent biochemical abnormalities also indicate that biological recovery may persist beyond symptom resolution, necessitating continued monitoring. Routine post-COVID biomarker assessment including inflammatory markers (IL-6, ferritin, CRP), lipid profile, hematological indices and micronutrient (vitamin D and B12) level assessments should be included in routine follow-up.

Moreover, raised inflammatory markers at ten months follow up and SARS-CoV-2 viral load during acute infection were also found to be associated with neuropsychiatry and musculoskeletal symptoms of Long COVID indicate the impact of viral load during acute infection on symptoms of Long COVID.

Future research using large scale, prospective, multi-center studies are essential to understand and identify physiological pathways involved in these individuals. These efforts are essential standardized clinical recommendations and optimizing long-term care plans for COVID-19 survivors.

## Limitations

The study was conducted in a single center restricting the general applicability of the results. Symptom assessment relied on patients themselves, raising concerns about reporting bias. We could not assess the symptoms of COVID-19 during acute infection due to possible recall bias. This could help in assessing if Long COVID symptoms correlate with previous symptoms or if they were more intense or prolonged. We did not have data regarding the biochemical markers of the participants during or before COVID-19 infection. Since pre-infection biochemical measurements were not available, we cannot exclude the possibility that some of the observed differences between Long COVID and control groups predated SARS-CoV-2 infection and were unrelated to COVID-19 itself.

Although our study identified statistically significant differences in several hematological and biochemical parameters, the clinical relevance of these findings must be interpreted with caution. Most of these parameters remained within established reference ranges hence should not be used as standalone markers of organ involvement. This suggests that isolated statistical significance may reflect normal physiological variation rather than pathophysiological effects of Long COVID. Emphasizing biologically plausible and consistently altered markers such as IL-6 and vitamin deficiencies provides a more meaningful framework for understanding persistent symptomatology and guiding future mechanistic and interventional studies.

The exclusion of individuals with pre-existing comorbidities such as diabetes, metabolic disorder, hypertension, may underestimate the true burden of Long COVID among a population where these conditions are common. Moreover, the laboratory parameters were measured from a single diagnostic facility, potentially introducing a selection bias.

## Supporting information

S1 Table Comparison of hematological and inflammatory parameters between controls and Long COVID participants. Hematological and inflammatory parameters were compared between controls (n = 123) and Long COVID participants (n = 177). Data is presented as median [IQR]. Group comparisons were performed using the Mann–Whitney U test. Statistical significance: *p < 0.05; **p < 0.01; ***p < 0.001. Abbreviations: RBC, red blood cell count; MCV, mean corpuscular volume; HCT, hematocrit; MCHC, mean corpuscular hemoglobin concentration; WBC, white blood cell count; IL-6, interleukin-6; IL-10, interleukin-10; CRP, C-reactive protein.
(DOCX)

S2 Table Sex-stratified comparison of hematological parameters between controls and Long COVID participants. Hematological parameters were analyzed separately in male and female participants. Data is presented as median [IQR]. Comparisons between controls and Long COVID participants within each sex were performed using the Mann–Whitney U test. A p value < 0.05 was considered statistically significant.
(DOCX)

S3 Table Comparison of biochemical and metabolic parameters between controls and Long COVID participants. Biochemical and metabolic parameters were compared between controls (n = 123) and Long COVID participants (n = 177). Data is presented as median [IQR]. Statistical comparisons were performed using the Mann–Whitney U test. Statistical significance: *p < 0.05; **p < 0.01; ***p < 0.001. Abbreviations: HDL, high-density lipoprotein; LDL, low-density lipoprotein; TRG, triglycerides; Vit D, vitamin D; B12, vitamin B12.
(DOCX)

S4 Table Comparison of laboratory parameters across symptom-based subgroups in Long COVID participants. Laboratory parameters were compared among Long COVID participants (n = 177) grouped by dominant clinical manifestations: neuropsychiatric (NEURO), musculoskeletal (MSK), respiratory (RESPO), reproductive (REPRO), and gastrointestinal (GIT). Data are presented as mean ± standard deviation (SD). Group comparisons were performed using one-way analysis of variance (ANOVA) followed by Tukey’s post hoc test. Statistical significance: *p < 0.05; **p < 0.01.
(DOCX)

S5 Table Multivariable logistic regression analysis of laboratory parameters associated with symptom clusters in Long COVID. Adjusted odds ratios (aOR) with 95% confidence intervals (CI) were estimated using multivariable logistic regression models to assess associations between laboratory parameters and symptom clusters. Analyses were adjusted for age and sex. Other symptoms include respiratory, reproductive, and gastrointestinal manifestations. Vitamin D deficiency was defined as serum 25-hydroxyvitamin D < 20 ng/mL. A p value < 0.05 was considered statistically significant.
(DOCX)
