# Supplementary material for: Acute SARS-CoV-2 viral load and systemic inflammation are associated with neuropsychiatric and musculoskeletal symptoms in long COVID
Source: PLoS One. 2026 Apr 15;21(4):e0346978. doi: 10.1371/journal.pone.0346978 (PMC13082598; doi:10.1371/journal.pone.0346978)
Supplement: S1 Table — Hematological and inflammatory parameters were compared between controls (n = 123) and Long COVID participants (n = 177). Data is presented as median [IQR]. Group comparisons were performed using the Mann–Whitney U test. Statistical significance: *p < 0.05; **p < 0.01; ***p < 0.001. Abbreviations: RBC, red blood cell count; MCV, mean corpuscular volume; HCT, hematocrit; MCHC, mean corpuscular hemoglobin concentration; WBC, white blood cell count; IL-6, interleukin-6; IL-10, interleukin-10; CRP, C-reactive protein. (DOCX) [file pone.0346978.s001.docx]

| **Laboratory Parameters** | **Controls (n=123)**  Median [IQR] | **Long COVID (n=177)**  Median [IQR] | **p value** |
| --- | --- | --- | --- |
| Hemoglobin (g/dL) | 13.00 [11.90–14.60] | 11.00 [10.90–13.00] | <0.001*** |
| RBC (×10^12^/L) | 5.10 [4.70–5.20] | 4.71 [4.40–5.60] | 0.034* |
| MCV (fL) | 87.00 [84.00–92.00] | 87.00 [80.00–92.00] | 0.724 |
| HCT (%) | 38.00 [36.20–40.00] | 38.00 [35.90–40.00] | 0.631 |
| MCHC (g/dL) | 35.00 [33.00–35.50] | 33.00 [31.50–34.00] | <0.001*** |
| WBC (×10^9^/L) | 5.50 [5.20–7.10] | 6.67 [4.90–6.60] | <0.001*** |
| Neutrophils (%) | 55.60 [48.10–66.10] | 55.90 [49.90–61.80] | 0.997 |
| Eosinophils (%) | 2.980 [1.70–3.80] | 3.10 [1.80–4.20] | 0.35 |
| Basophils (%) | 0.47 [0.30–0.60] | 0.40 [0.20–0.60] | 0.896 |
| Lymphocytes (%) | 31.90 [24.50–36.20] | 28.87 [20.30–30.70] | 0.001** |
| Monocytes (%) | 4.70 [3.20–7.10] | 4.10 [2.90–5.90] | 0.069 |
| IL-6 (pg/mL) | 0.70 [0.40–1.00] | 3.30 [1.70–4.20] | < 0.001*** |
| Il-10 (pg/mL) | 4.79 [4.40–5.60] | 4.70 [4.30–5.10] | 0.866 |
| CRP (mg/L) | 0.71 [0.60–1.00] | 0.98 [0.60–1.00] | 0.811 |
| Ferritin (ng/mL) | 121.00 [110.00–160.00] | 127.00 [100.00–160.00] | 0.013* |

**S1 table: S1 Table. Comparison of hematological and inflammatory parameters between controls and Long COVID participants.**

Hematological and inflammatory parameters were compared between controls (n = 123) and Long COVID participants (n = 177). Data is presented as median [IQR]. Group comparisons were performed using the Mann–Whitney U test. Statistical significance: *p < 0.05; **p < 0.01; ***p < 0.001.

Abbreviations: RBC, red blood cell count; MCV, mean corpuscular volume; HCT, hematocrit; MCHC, mean corpuscular hemoglobin concentration; WBC, white blood cell count; IL-6, interleukin-6; IL-10, interleukin-10; CRP, C-reactive protein.
