# Supplementary material for: Acute SARS-CoV-2 viral load and systemic inflammation are associated with neuropsychiatric and musculoskeletal symptoms in long COVID
Source: PLoS One. 2026 Apr 15;21(4):e0346978. doi: 10.1371/journal.pone.0346978 (PMC13082598; doi:10.1371/journal.pone.0346978)
Supplement: S2 Table — Hematological parameters were analyzed separately in male and female participants. Data is presented as median [IQR]. Comparisons between controls and Long COVID participants within each sex were performed using the Mann–Whitney U test. A p value < 0.05 was considered statistically significant. (DOCX) [file pone.0346978.s002.docx]

**S2 Table. Sex-stratified comparison of hematological parameters between controls and Long COVID participants.**

| **Parameter** | **Males (n=157)** | | **p value** | **Females (n=143)** | | **p value** |
| --- | --- | --- | --- | --- | --- | --- |
|  | **Controls (n=62)** | **Long COVID (n=95)** |  | **Controls (n=82)** | **Long COVID (n=61)** |  |
| Hemoglobin (g/dL) | 13.8 [11.54-15.89] | 12.4 [11.2-12.9] | 0.0031 | 12.4 [10.68-13.21] | 10.9 [10.32-11.98] | <0.001 |
| RBC (×10⁶/µL) | 5.2 [5.1-5.9] | 4.8 [4.1-5.1] | <0.001 | 4.7 [4.2-5.1[ | 4.4 [4.1-4.8] | 0.002 |
| Hematocrit (%) | 40.2 [38.1-42.5] | 38.6 [35.5-39.2] | 0.213 | 37.8 [35.4-38.5] | 36.9 [33.5-38.6] | 0.756 |

Hematological parameters were analyzed separately in male and female participants. Data is presented as median [IQR]. Comparisons between controls and Long COVID participants within each sex were performed using the Mann–Whitney U test. A p value < 0.05 was considered statistically significant.
