# Supplementary material for: Acute SARS-CoV-2 viral load and systemic inflammation are associated with neuropsychiatric and musculoskeletal symptoms in long COVID
Source: PLoS One. 2026 Apr 15;21(4):e0346978. doi: 10.1371/journal.pone.0346978 (PMC13082598; doi:10.1371/journal.pone.0346978)
Supplement: S3 Table — Biochemical and metabolic parameters were compared between controls (n = 123) and Long COVID participants (n = 177). Data is presented as median [IQR]. Statistical comparisons were performed using the Mann–Whitney U test. Statistical significance: *p < 0.05; **p < 0.01; ***p < 0.001. Abbreviations: HDL, high-density lipoprotein; LDL, low-density lipoprotein; TRG, triglycerides; Vit D, vitamin D; B12, vitamin B12. (DOCX) [file pone.0346978.s003.docx]

**S3 Table. Comparison of biochemical and metabolic parameters between controls and Long COVID participants.**

| **Laboratory Parameters** | **Controls (n=123)**  Median [IQR] | **Long COVID (n=177)**  Median [IQR] | **p value** |
| --- | --- | --- | --- |
| Total Cholesterol (mg/L) | 187.00 [176.00–200.00] | 186.00 [176.00–199.00] | 0.963 |
| HDL (mg/L) | 52.00 [48.00–57.00] | 54.00 [49.00–58.00] | 0.337 |
| LDL (mg/L) | 87.00 [80.00–96.00] | 84.00 [87.00–103.00] | 0.091 |
| TRG (mg/L) | 89.00 [83.00–98.00] | 89.00 [81.00–99.00] | 0.959 |
| UREA (mg/L) | 18.76 [13.00–21.00] | 19.3 [17.00–25.00] | 0.56 |
| Creatinine (mg/L) | 0.890 [0.70–1.00] | 0.90 [0.80–1.00] | 0.11 |
| Sodium (mEq/L) | 138.00 [135.00–140.00] | 137.00 [135.00–139.00] | 0.238 |
| Potassium (mEq/L) | 3.60 [3.50–3.80] | 3.60 [3.40–3.80] | 0.563 |
| Chloride (mEq/L) | 101.00 [98.00–108.00] | 99.90 [96.00–107.00] | 0.095 |
| Bicarbonate (mEq/L) | 24.00 [23.00–26.00] | 25.00 [23.00–27.00] | 0.263 |
| Vit D (ng/mL) | 24.00 [20.00–28.00] | 19.80 [17.60–24.60] | 0.002** |
| B12 (pg/mL) | 395.00 [345.00–450.00] | 347.00 [299.00–455.00] | 0.014* |

Biochemical and metabolic parameters were compared between controls (n = 123) and Long COVID participants (n = 177). Data is presented as median [IQR]. Statistical comparisons were performed using the Mann–Whitney U test. Statistical significance: *p < 0.05; **p < 0.01; ***p < 0.001.

Abbreviations: HDL, high-density lipoprotein; LDL, low-density lipoprotein; TRG, triglycerides; Vit D, vitamin D; B12, vitamin B12.
