# Supplementary material for: Acute SARS-CoV-2 viral load and systemic inflammation are associated with neuropsychiatric and musculoskeletal symptoms in long COVID
Source: PLoS One. 2026 Apr 15;21(4):e0346978. doi: 10.1371/journal.pone.0346978 (PMC13082598; doi:10.1371/journal.pone.0346978)
Supplement: S4 Table — Laboratory parameters were compared among Long COVID participants (n = 177) grouped by dominant clinical manifestations: neuropsychiatric (NEURO), musculoskeletal (MSK), respiratory (RESPO), reproductive (REPRO), and gastrointestinal (GIT). Data are presented as mean ± standard deviation (SD). Group comparisons were performed using one-way analysis of variance (ANOVA) followed by Tukey’s post hoc test. Statistical significance: *p < 0.05; **p < 0.01. (DOCX) [file pone.0346978.s004.docx]

**S4 Table. Comparison of laboratory parameters across symptom-based subgroups in Long COVID participants.**

| Lab parameter | Total Mean  (SD) | NEURO (n=62) | MSK (n=57) | RESPO (n=24) | REPRO (n=24) | GIT (n=10) | *p* value |
| --- | --- | --- | --- | --- | --- | --- | --- |
| Lymphocytes (%) | 30.5 ± 6.0 | 33.12 ± 6.3 | 32.11 ± 6.1 | 29.87 ± 5.7 | 27.78 ± 5.6 | 28.97 ± 5.8 | 0.012* |
| IL-6 (pg/mL) | 4.12 ± 2.1 | 5.89 ± 2.4 | 5.11 ± 2.3 | 3.90 ± 1.8 | 3.60 ± 1.7 | 3.43 ± 1.6 | 0.003** |
| Ferritin (ng/mL) | 143 ± 48 | 146.12 ± 49 | 144.23 ± 46 | 138.13 ± 45 | 135.0 ± 44 | 137.9 ± 46 | 0.0012** |
| Vit D (ng/mL) | 18.45 ± 7.0 | 13.34 ± 6.1 | 14.11 ± 6.2 | 19.23 ± 7.4 | 17.98 ± 6.7 | 16.11 ± 6.5 | 0.005** |
| Vit B12 (pg/mL) | 361 ± 92 | 347.41 ± 88 | 332.78 ± 84 | 399.34 ± 101 | 367.94 ± 95 | 360.83 ± 91 | 0.0045** |

Laboratory parameters were compared among Long COVID participants (n = 177) grouped by dominant clinical manifestations: neuropsychiatric (NEURO), musculoskeletal (MSK), respiratory (RESPO), reproductive (REPRO), and gastrointestinal (GIT). Data are presented as mean ± standard deviation (SD). Group comparisons were performed using one-way analysis of variance (ANOVA) followed by Tukey’s post hoc test. Statistical significance: *p < 0.05; **p < 0.01.
