# Supplementary material for: Acute SARS-CoV-2 viral load and systemic inflammation are associated with neuropsychiatric and musculoskeletal symptoms in long COVID
Source: PLoS One. 2026 Apr 15;21(4):e0346978. doi: 10.1371/journal.pone.0346978 (PMC13082598; doi:10.1371/journal.pone.0346978)
Supplement: S5 Table — Adjusted odds ratios (aOR) with 95% confidence intervals (CI) were estimated using multivariable logistic regression models to assess associations between laboratory parameters and symptom clusters. Analyses were adjusted for age and sex. Other symptoms include respiratory, reproductive, and gastrointestinal manifestations. Vitamin D deficiency was defined as serum 25-hydroxyvitamin D < 20 ng/mL. A p value < 0.05 was considered statistically significant. (DOCX) [file pone.0346978.s005.docx]

| Lab parameter | Neuropsychiatric symptoms | | | Musculoskeletal symptoms | | | Other symptoms* | | |
| --- | --- | --- | --- | --- | --- | --- | --- | --- | --- |
|  | Adjusted OR | 95% CI | p value | Adjusted OR | 95% CI | p value | Adjusted OR | 95% CI | p value |
| Lymphocytes (%) | 1.19 | 1.12-1.51 | **0.02** | 0.78 | 0.66-1.10 | 0.12 | 0.37 | 0.12–1.19 | 0.096 |
| IL-6 (pg/mL) | 1.16 | 1.10–1.86 | **0.030** | 0.94 | 0.72-0.99 | **0.036** | 0.64 | 0.40–1.01 | 0.056 |
| Ferritin (ng/mL) | 1.42 | 1.10-1.53 | **0.002** | 0.98 | 0.97-0.99 | **0.006** | 0.98 | 0.76–1.93 | 0.25 |
| Vit D (<20ng/dl) | 1.45 | 1.22-2.01 | **0.003** | 2.3 | 1.2–4.5 | **0.001** | 1.02 | 1.00 – 1.03 | **0.04** |
| Vit B12 (pg/mL) | 0.86 | 0.70-1.10 | 0.07 | 1.1 | 1.00 – 1.23 | 0.05 | 1.40 | 0.46–4.22 | 0.55 |

**S5 Table. Multivariable logistic regression analysis of laboratory parameters associated with symptom clusters in Long COVID.**

Adjusted odds ratios (aOR) with 95% confidence intervals (CI) were estimated using multivariable logistic regression models to assess associations between laboratory parameters and symptom clusters. Analyses were adjusted for age and sex. Other symptoms include respiratory, reproductive, and gastrointestinal manifestations. Vitamin D deficiency was defined as serum 25-hydroxyvitamin D <20 ng/mL. A p value < 0.05 was considered statistically significant.
